# Advances in Cardiotoxicity Induced by Altered Mitochondrial Dynamics and Mitophagy

**DOI:** 10.3389/fcvm.2021.739095

**Published:** 2021-09-20

**Authors:** Yiyuan Yin, Haitao Shen

**Affiliations:** Department of Emergency Medicine, ShengJing Hospital of China Medical University, Shenyang, China

**Keywords:** mitochondrial dynamics, cardiotoxicity, mitophagy, mitochondrial fission, mitochondrial fusion

## Abstract

Mitochondria are the most abundant organelles in cardiac cells, and are essential to maintain the normal cardiac function, which requires mitochondrial dynamics and mitophagy to ensure the stability of mitochondrial quantity and quality. When mitochondria are affected by continuous injury factors, the balance between mitochondrial dynamics and mitophagy is broken. Aging and damaged mitochondria cannot be completely removed in cardiac cells, resulting in energy supply disorder and accumulation of toxic substances in cardiac cells, resulting in cardiac damage and cardiotoxicity. This paper summarizes the specific underlying mechanisms by which various adverse factors interfere with mitochondrial dynamics and mitophagy to produce cardiotoxicity and emphasizes the crucial role of oxidative stress in mitophagy. This review aims to provide fresh ideas for the prevention and treatment of cardiotoxicity induced by altered mitochondrial dynamics and mitophagy.

## Introduction

As the body's “power plant,” heart is the body's largest oxygen and energy consumption organ. Therefore, mitochondria, as the core organelles of oxidative phosphorylation, play an important role in maintaining cardiac homeostasis. Under normal conditions, cardiac cells regulate the dynamic balance of mitochondria through a variety of signal pathways, remove damaged mitochondria through the process of mitochondrial fission, fusion and autophagy, and maintain the normal cardiac function. However, injury factors such as hypoxia, oxidative stress, poisoning, and hyperglycemia can cause abnormalities in mitochondrial dynamics and mitophagy, resulting in cardiotoxicity. Therefore, interventional treatment for different injury factors is of great significance for improving cardiotoxicity induced by altered mitochondrial dynamics and mitophagy.

## Concept of Mitochondrial Dynamics and Mitophagy

Mitochondria are critical organelles of eclectic cells and can reach 25–35% of cell volume (1–3). They have a phospholipid bimolecular membrane structure and play a crucial role in maintaining normal functionality in cells and metabolizing steady state. Moreover, they are also the primary locations for the oxidative metabolism of cells. Also, mitochondria have a mediated effect on cell proliferation or apoptosis, regulation of nuclear gene expression, and innate immunity (4, 5). Under normal physiological conditions, mitochondria produce ATP through the tricarboxylic acid cycle to meet the energy needs of the heart (6–8). It is not only the power plant of the cell, but also the center of signal transmission including calcium homeostasis (9, 10), which ensure the normal operation of the mitochondrial electron transport chain to maintain the normal cardiac function (11, 12). In the electron transport chain, premature leakage of electrons will lead to the production of physiological reactive oxygen species (ROS), and a small amount of ROS can be decomposed by superoxide dismutase (SOD) and glutathione (GSH) in the mitochondria (9, 10, 13). When the mitochondria is in an abnormal state, the tricarboxylic acid cycle and calcium homeostasis are destroyed, and the mitochondrial membrane potential dissipation in turn leads to the disorder of the electron transport chain and the accumulation of ROS (14, 15). The original mitochondrial quality of cardiac cells cannot maintain the normal function of cardiac cells, resulting in cardiac dysfunction and cardiotoxicity (6, 16, 17).

Although mitochondria are usually described as independent organelles, they actually form a dynamic equilibrium network maintained by mitochondrial dynamics, which is essential for maintaining normal cell metabolism. In mitochondrial structures, the outer mitochondrial membranes (OMM) comprises a relatively smooth lipid double layer, and the inner mitochondrial membrane (IMM) folds inwards to form a structure called argon (18). The fission of membranes and outer membranes in mitochondria is a critical event in mitochondrial fission; it is a process that divides a single mitochondrion into two mitochondria, guided by a dynamin-related protein 1 (DRP1) (19). Mitochondrial fusion is divided into outer membrane fusion and endometrial fusion, the balance of which determines the connectivity of the network (20).

Mitophagy is the process that identifies damaged mitochondria in cells, which in turn binds to autophagy-related proteins to create autophagic small bodies. These bodies are degraded by fusion with lysosomes (21). Generally, mitochondria are abundant in cardiac cells, making the cardiac cells more sensitive to alterations in mitochondrial functionality (22). Under normal circumstances, a certain mitophagy level promptly removes damage to aging mitochondria and metabolic toxic substances, promotes mitochondrial renewal, and ensures the survival of cells (23–25).

## The Physiological State of Mitochondrial Dynamics and Mitophagy

Cardiotoxicity is caused by altered mitochondrial dynamics and mitophagy (26, 27). The cardiac cells can remove dysfunctional mitochondria through mitochondrial fission, fusion, and autophagy. The process has a direct regulatory effect on the quantity and quality of the cardiac mitochondria. Thus, it ensures the stability of the inner environment of cardiac cells (28–32). Under normal physiological conditions, mitochondria are constantly updated to sustain healthy cardiac functionality. Moreover, it can promote the formation of new mitochondria and maintain the cardiac continuous contraction. At this stage, the cardiac can promptly remove damaged mitochondria through fission, fusion, and autophagy, and facilitate the recovery of effective cellular components, such as proteins, deoxyribonucleic acid (DNA), etc., to ensure the normal metabolism of updated cells, thus compensate to ensure the nominal function of mitochondria to maintain the cardiac continuous contraction state (33).

It is generally believed that fission and fusion are carried out at the same time and are dynamically balanced, and fission is often regarded as a prerequisite for mitophagy (34–37). Parkin, the key protein of mitophagy, can induce ubiquitination or degradation of MFN1/2, thereby inhibiting mitochondrial fusion (38, 39). The significance of mitophagy for fusion is that when damaged mitochondria fuse with healthy mitochondria, a larger damaged mitochondria will be formed, which can activate mitophagy and maintain mitochondrial homeostasis (34, 40–42). In mitochondrial fission, fusion and mitophagy, mitochondrial autophagy plays a central role (34).

### Mitochondrial Fission

Mitochondrial fission is divided into the fission of membranes and outer membranes in mitochondria and is regulated by DRP1 (43, 44). DRP1 is classified as a homologous protein of guanosine triphosphate (GTP) hydrolyzed enzyme (GTPase) power protein. It has an active role in endocytosis and is a key regulatory factor in mitochondrial fission, primarily located in cell pulp (20, 45, 46).

The serine 637 (S637) phosphorylation of DRP1 inhibits the translocation of mitochondria DRP1 and its GTPase activity. Meanwhile, serine 616 (S616) phosphorylation elevates the DRP1 activity, which splits the mitochondria. During the fission process, mitochondrial fission 1 (FIS1) protein, mitochondrial fission factor (MFF), 49 kDa mitochondrial dynamic protein (MiD49), and 51 kDa mitochondrial kinetic protein (MiD51) induce DRP1 phosphorylation to recruit DRP1 into the mitochondrial outer membrane. Afterward, the DRP1 oligopoly reaction at the fission point of the OMM self-assembles to create a spiral structure. It forms a cleavage ring that shrinks and shears the mitochondrial outer and inner membranes and breaking the mitochondria (47–54). Notably, FIS1 is distributed throughout the outer membrane, while MFF is dotted, showing a stronger interaction with DRP1 than FIS1. FIS1 and MFF can independently promote the collection and oligopoly of the mitochondrial outer membrane, yet, MFF plays a more critical role. Besides, in the absence of MFF and FIS1, MiD49 and MiD51 can recruit DRP1 to the mitochondria (49).

Furthermore, cyclase-associated protein (CAP) are recently discovered split-promoting proteins that induce the oligomerization of DRP1 and the expression of FIS1, which promotes DRP1-mediated mitochondrial fission (55).

### Mitochondrial Fusion

Mitochondrial fusion is divided into outer membrane fusion and endometrial fusion. It is regulated by a variety of proteins, including mitofusin (MFN) in the outer membrane of mitochondria and optic atrophy protein 1 (OPA1) (44, 45, 49, 51, 56–61). Among them, mitofusin 1 (MFN1) and mitofusin 2 (MFN2) regulate mitochondrial outer membrane fusion. MFN1 positioned mitochondria and MFN2 positioned mitochondria and endoplasmic reticulum. MFN1 and MFN2 form a stable homologous dimer through their GTPase domain. Next, hydrolyzed GTP and the outer membrane of the two mitochondria are combined and fused, which is critical for outer membrane fusion (49, 52, 54, 56).

The OPA1 regulates the fusion of the IMM. OPA1 is treated with mitochondrial processing peptide (MPP) enzyme to produce a long-form OPA1 (L-OPA1) of membrane binding, and then positioned as intermembrane space AAA (i-AAA) protease in the membrane of the mitochondria IMM peptide enzymes and mitochondrial AAA (m-AAA) protease are further cut into short -form OPA1 (S-OPA1). Afterward, the mitochondrial membranes are arranged into two layers of film while maintaining the fidelity of the mitochondrial crucible structure and promoting endometrial fusion (49, 58, 62).

### Mitophagy

Mitophagy is an autophagy process that is regulated by several mechanisms and protein molecules. However, it is different than ordinary autophagy and is highly selective (63, 64). Presently, three major pathways can induce and activate mitophagy: PTEN-induced kinase 1 (PINK1)-Parkin signaling pathway, BCL2 interacting protein 3 (BNIP3)/NIP3-like protein X (NIX) pathway, and the FUN14 domain containing 1 (FUNDC1) signaling pathway. Out of the three, the PINK1-Parkin signaling pathway is the most characteristic and significant autophagy pathway (65–67). In mitophagy, different pathways cooperate and coordinate to sustain the normal functionality in cells.

#### The PINK1-Parkin Signaling Pathway

PINK1 is a mitochondrial serine-threonine protein kinase. When the mitochondrial membrane potential decreases, the PINK1-Parkin signaling pathway PINK1 aggregates in the mitochondrial membrane's outer membrane and activates Parkin on damaged mitochondria (68–76). Generally, PINK1 is less expressed. It enters the mitochondria, anchors the mitochondrial intima through the mediation of outer membrane-related proteins. Under external stimulation or pathological conditions, mitochondrial depolarization can cause PINK1 translocation to mitochondria's outer membrane. Afterward, it catalyzes ubiquitin phosphorylation to activate the Parkin receptor binding Parkin and initiate mitophagy (30, 70, 77–85). Moreover, the PINK1-Parkin pathway is also the primary mechanism of Zinc induced mitophagy (86).

#### BNIP3/NIX Pathway

BNIP3 (also known as NIX) is a member of the Bcl-2 protein family. It is a form of a mitochondrial outer membrane protein with a biphasic effect. The phosphorylation of their microtubule-associated protein 1A/1B-light chain 3 (LC3)-interacting region (LIR) binds to LC3-phosphatidylethanolamine conjugate light chain 3 (LC3II), which is involved in mitophagy and plays a significant role in myocardial mitochondrial regeneration (24, 87–89). The hypoxia inducible factor 1 subunit alpha (HIF-1α) can bind to the BNIP3 promoter to induce BNIP3, and BNIP3 expression can also promote PINK1 translocation, and then induce mitophagy (90). Reportedly, the cardiac dual-specificity phosphatase-1 (DUSP1) also induces BNIP3 expression and promotes mitophagy (91).

#### FUNDC1 Signaling Pathway

The FUNDC1 is a highly conserved mitochondrial outer membrane protein. Similar to BNIP3/NIX, it directly interacts with LC3 through the N end, mediating hypoxia-induced mitophagy, which is widely expressed in various cells, tissues, and organs, particularly heart (63, 79, 88, 90, 92, 93). Under normal oxygen conditions, FUNDC1 phosphorylated by semi refined carrageenan (SRC) kinases and Casein kinase II (CK2) decreases their affinity to LC3, which effectively inhibits mitophagy. FUNDC1 was dephosphorylated by serine 13-position phosphatase, such as PGAM family member 5 (PGAM5), triggering its association with LC3, thereby enhancing mitophagy (23, 88, 93).

### Biogenesis

Mitochondrial biogenesis is also considered to be an important factor in maintaining mitochondrial homeostasis. It is a complex process involving the synthesis of mitochondrial inner and outer membranes and mitochondrial-encoded proteins, the synthesis and input of mitochondrial-encoded proteins, and the replication of mitochondrial DNA (mtDNA) (94–97), which is mainly regulated by PPARG Coactivator 1 Alpha (PGC-1α) and Nuclear Respiratory Factor 1 (NRF1), and can be defined as “the process of producing new components of the mitochondrial network” (98–100). Some scholars evaluate the biogenesis of mitochondria by measuring the rate of mitochondrial protein synthesis. Mitochondrial biogenesis and mitophagy coordinately regulate the molecular mechanism of mitochondrial homeostasis (101–104). On the one hand, the process of mitochondrial biogenesis is accompanied by mitophagy, on the other hand, abnormal mitophagy can feedback and inhibit mitochondrial biogenesis, and the PGC-1α-NRF1-FUNDC1 pathway plays a key role in it, cooperating to maintain the quality and quantity of mitochondria (96, 97, 105, 106).

## The Pathophysiological State of Mitochondrial Dynamics and Mitophagy

**Figure 1:**
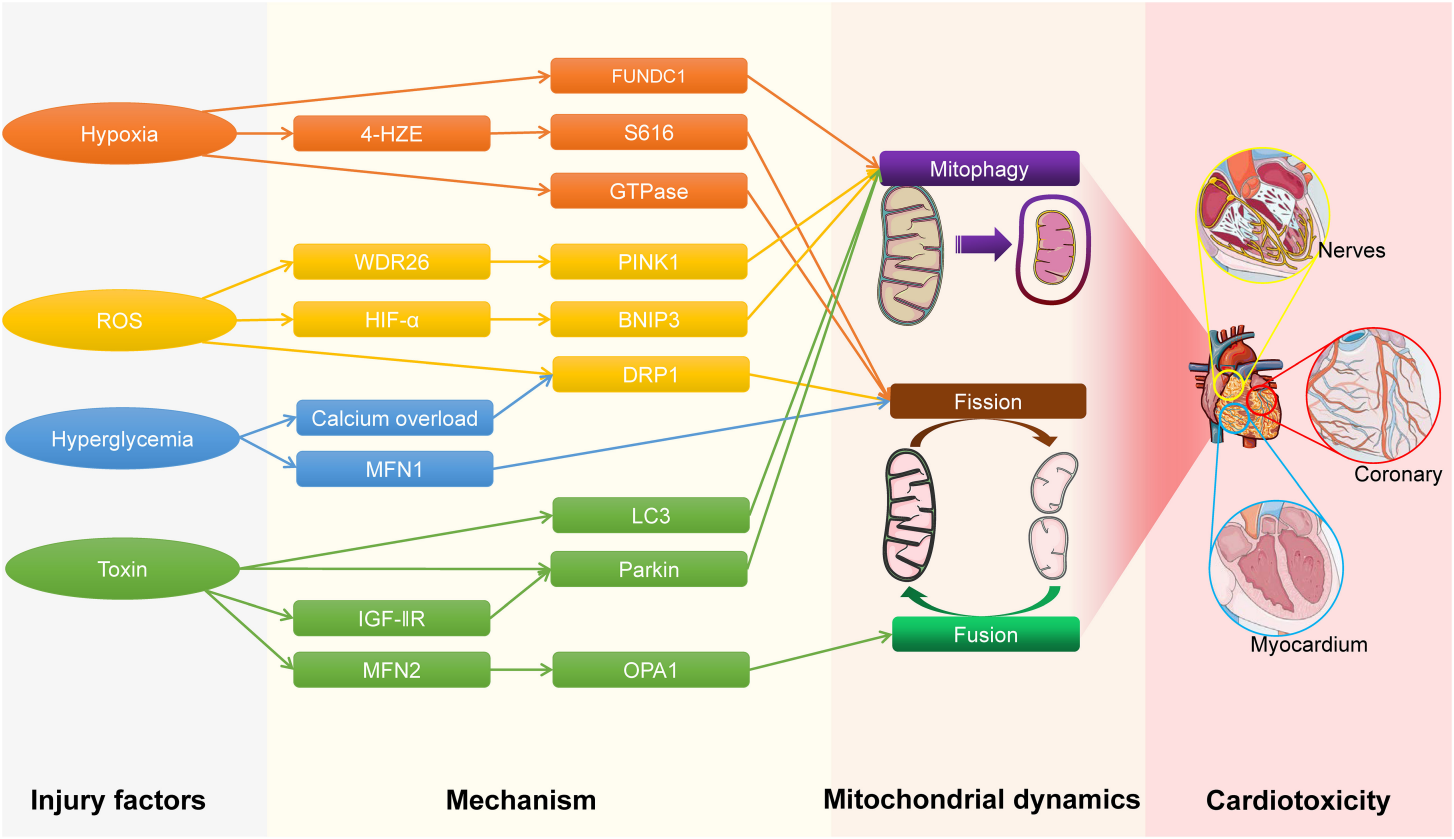
The process of cardiotoxicity caused by different injury factors.

When cardiac cells are stimulated by mechanical traction, ischemia-reperfusion injury, and oxidative stress, they can cause changes in the shape, structure and function of the heart (107–110). Multiple signal pathways are activated when the heart is stimulated by pathogenic factors, such as mitogen-activated protein kinase signaling pathway, calcineurin (CaN) signaling pathways, protein kinase A signaling pathways and angiotensin type I receptors cause calcium homeostasis to be destroyed and calcium overload, which ultimately leads to cardiac pathophysiological changes (111–114). When excessive or continuous stress acts on the heart, the mitochondrial energy metabolism function and quality control system are seriously disturbed, which exceeds the self-regulation range of mitochondrial dynamics and mitophagy. On the one hand, it causes the energy metabolism of cardiac cells to become impaired, on the other hand, the mtDNA and ROS released by damaged mitochondria accumulate to reach a toxic concentration, which together lead to cardiotoxicity (25, 66, 115–117), the process of cardiotoxicity caused by different injury factors is shown in Figure 1. Cardiotoxicity refers to cardiac damage caused by excessive accumulation of endogenous or exogenous substances to reach a toxic concentration (118). Generally speaking, cardiotoxicity can cause cardiac electrophysiological dysfunction or myocardial damage (119). Moreover, mitochondrial dynamics play a vital role in the onset of nervous system diseases, implying that mitochondrial dynamics disorders may have damaging effects on cardiac neuronal cells (120).

In addition, among smoking and obesity people, the cardiovascular morbidity has increased significantly (121–126). It has been reported that smoking and obesity can cause abnormal mitochondrial dynamics and mitophagy (127–131). Therefore, smoking and obesity may also lead to mitochondrial damage, which in turn causes cardiac dysfunction, leading to cardiotoxicity.

### Hypoxia

Generally, hypoxia refers to any kind of physiological oxygen deficiency or tissue oxygen demand deficiency state and the integration of local responses defines hypoxia as a paradigm of reactions affecting the entire body (86, 132, 133). Studies have shown that inhibiting the breathing of rats caused obvious cardiotoxicity (79, 134–138).

#### Mitochondrial Fission, Fusion, and Hypoxia

During mitochondrial fission, lack of oxygen can increase the production of 4-hydroxyethyl ether (4-HNE) to promote the S616 phosphorylation of DRP1 to induce mitochondrial fission. Besides, in the presence of histone deacetylase 6 (HDAC6), hypoxia can promote mitochondrial fusion by inducing the binding of HDAC6 with MFN2, causing mitochondrial dysfunction (139–141).

#### Mitophagy and Hypoxia

Hypoxia can be specifically activated by FUNDC1; under normal conditions, FUNDC1 is highly conserved and stable in mitochondria's outer membrane. During hypoxia, it is FUNDC1 dephosphorylated by 13 phosphatases (such as PGAM5) of serine, triggering its binding to LC3 and improving mitophagy activity. It removes damaged mitochondria (23, 79, 88, 92, 93, 142–144). Hypoxia can activate poly (ADP-ribose) polymerase (PARP), promoting mitophagy by regulating mitochondrial membrane potential and inducing cardiomyocyte apoptosis, ROS is central for PARP mediated mitochondrial membrane potential (ΔΨm) decline, and inhibited PARP can reduce the production after injury (80). Moreover, the activation of FUNDC1 is vital in platelet aggregation. Past studies have demonstrated that lacking the FUNDC1 gene can make the mitochondrial function of blood platelet disordered. In long-term hypoxia, it will eventually form a microthrombus and lead to cardiac microvascular structure destruction (79, 145).

### Oxidative Stress

Oxidative stress (OS) is a state of imbalance between oxidation and antioxidant effect in the body. It produces several destructive products, such as ROS, which has an adverse impact on the body and is often considered to be a crucial factor that leads to aging and disease (146, 147). OS is caused by the imbalance between ROS and endogenous antioxidants in response to injury, which can lead to cardiotoxicity (148). ROS is a collective common term that includes highly oxidative radicals such as hydroxyl (OH-) and superoxide (O2•-) radicals, and non-radical species such as hydrogen peroxide (H2O2) (149–151). Antioxidants in the mitochondria, such as superoxide dismutase (SOD) and glutathione (GSH), will rapidly degrade or sequester O2·-, thereby reducing reactivity (152–154). Due to the high concentration of mitochondria in myocardial tissue, reduced mitochondrial antioxidant capacity results in cardiac dysfunction (155–157). In addition, ROS is involved in a series of vascular diseases associated with the functional properties of the endothelial cell barrier (158–160).

Reportedly, ROS can significantly promote the activity of DRP1 to increase the mitochondrial fission frequency, resulting in mitochondrial dysfunction. Oxidative stress can significantly increase the expression of WD repeat domain 26 (WDR26) protein, which is a critical medium for PINK1-Parkin signaling pathways to induce cell mitophagy and depolarize mitochondria by elevating the mitochondrial membrane potential, causing PINK1 to transpose, which in turn catalyzes ubiquitin phosphorylation to activate the Parkin receptor (70, 78–80, 161). Parkin is dependent on p53, it triggers mitophagy through autophagy small body lysosome pathways and then degrades through autophagy-lysosome pathways. Moreover, oxidative stress could also lead to an extended opening time for mitochondrial permeability transition pore (mPTP), releasing apoptosis factors such as cytochrome c into the matrix, damaging cells (162–164). Meanwhile, ROS activates multiple inflammatory pathways such as NLR family pyrin domain containing 3 (NLRP3)-mediated inflammatory responses. And the inhibition of mitophagy further aggravates these inflammatory responses and exacerbates damage (165). During myocardial ischemic re-perfusion injury (MIRI), the cell ischemia hypoxia activates PINK1/Parkin-mediated mitophagy and then removes the defective mitochondria. Afterward, restores the intracellular steady-state to offset the damage inflicted by hypoxia. In the case where Parkin is lacking, it will further aggravate ischemia re-perfusion damage and inflict damage to the heart (78, 166–170). Uncoupling protein 2 (UCP2) and vitamin D interferes with abnormal mitophagy to protect the damaged cardiac from ischemic re-injection (171, 172). Moreover, oxidative stress reactions can also activate mitophagy through BNIP3/NIX and ROS promotes BNIP3 expression by activating the HIF-1α, which subsequentially induces mitophagy (89, 90). Reportedly, the oxidative stress response is a crucial cause of mitophagy disorders in diabetic patients (24). In addition, membrane associated Ring-CH-Type Finger 5 (MARCHF5) and cellular communication network factor 1 (CCN1/Cyr61) are protein molecules located in the mitochondrial outer membrane, these proteins also play a vital role in the autophagy process of mitochondria, reducing expression during oxidative stress, and further inhibits mitophagy (173, 174).

### Hyperglycemia

Studies have shown that hyperglycemia can increase the opening of mPTP by causing mitochondrial rupture and stimulating the generation of ROS, leading to the release of cytochrome c into the cytoplasm to activate the NLRP3 inflammasome (175–177). Subsequently, NLRP3 activates downstream nuclear factors to cause the release of inflammatory factors such as TNF-α and IL-6 further promotes the occurrence of inflammation, which well-explains the pathogenesis of diabetic cardiomyopathy (178–180).

Hyperglycemia causes calcium overload by activating the ORAI calcium release-activated calcium modulator 1 (ORAI1) channel-mediated Ca^2+^ internal flow pathway. It would induce S616 phosphorylation to further advance the expression of DRP1 and inhibit the MFN1 gene expression, as well as promote mitochondria fission, resulting in mitochondrial dysfunction (53, 181, 182). Moreover, protein kinase A activity is significantly inhibited at low glucose levels, enhancing the positioning capacity of DRP1 on the outer membrane of the mitochondria, which significantly increases the rate of mitochondrial fission (183). Past studies have established that hunger or reduced insulin signals are a strong trigger for autophagy (92, 184, 185). Hyperglycemia can induce myocardial mitochondria division but inhibit mitophagy, causing the accumulation of functionally impaired mitochondria (57, 186, 187). Additionally, DRP1 and ROS have mutually reinforcing associations (188). As a result, oxidative stress reactions increase and ROS accumulates during hyperglycemia conditions, which further damages cardiac cells (24, 189).

### Poisoning

Poisoning refers to the systemic damage caused by the poisoning amount of harmful substances after entering the human body. Cardiotoxicity caused by drugs is divided into type I cardiotoxicity and type II cardiotoxicity (190–193). Among them, type I cardiotoxicity is associated with irreversible cardiac cell injury and is typically caused by anthracyclines and conventional chemotherapeutic agents, such as doxorubicin (DOX), daunorubicin, taxane and so on (194–196). Type II cardiotoxicity, associated with reversible myocardial dysfunction, is generally caused by biologicals and targeted drugs, such as trastuzumab, pertuzumab, azidothymidine, sumatinib, cloflupine, and cocaine, ethanol, etc (197, 198). The above-mentioned drugs can cause cardiotoxicity by interfering with mitochondrial dynamics and mitophagy, and ROS plays an important role in this process (26, 199–202). Studies have shown that anthracyclines such as doxorubicin and daunorubicin accumulate in the heart by binding to cardiolipin in the inner mitochondrial membrane (198). Anthracyclines binds with high affinity to the mitochondrial phospholipid cardiolipin, inhibits its function, stimulates ROS production, inhibits oxidative phosphorylation, and causes mitochondrial DNA damage. These events result in mitochondrial defects, leading to the opening of mPTP and the activation of cell death pathways, which precipitate myocardial dysfunction (27, 203, 204). In addition, recent experimental studies have found mitochondrial iron accumulation following doxorubicin to be the mediator of doxorubicin cardiotoxicity from redox cycling and oxidative injury. ABCB8, a mitochondrial transport protein facilitates the export of iron from the mitochondria. Doxorubicin reduces ABCB8 transporter in the mitochondria. Overexpression of ABCB8 protein or administration of dexrazoxane, an iron chelator reverses the anthracycline-induced mitochondrial iron overload and oxidative injury. It has been reported that the expression of TNF-α and IL-6 in the myocardial tissue and H9C2 cells treated with DOX increased significantly (1, 198, 205–208). Taxane further inhibits mitophagy by interfering with the normal microtubular transport function in the cardiomyocytes (26, 153, 198).

Unlike anthracyclines, trastuzumab induced left ventricular dysfunction (LVD) and congestive heart failure (CHF) are mostly reversible upon its discontinuation. At a molecular level, trastuzumab binds to the extracellular domain 4 of HER2 receptor, which prevents HER2 dimerization, activation and downstream signaling (190, 194–196). It may induce the occurrence of oxidative stress, which can also lead to the opening of mPTP and the activation of cell death pathways, leading to cardiac dysfunction (192, 209, 210). There are reports that drugs that cause type II cardiotoxicity can also enhance anthracycline cardiotoxicity, such as azidothymidine and rosiglitazone (193, 198, 211). In addition, there are some antidepressants and excessive metal elements, such as cloflupine, cocaine, antimony, mercury and so on (197, 212).

#### Mitochondrial Fission, Fusion and Poisoning

Poisoning can change the mitochondrial dynamically regulated protein expression. On the one hand, poisoning induces the expression of MFN2 and OPA1 in the cardiac tissue, which increases the mitochondrial length and organelle aspect ratio and excessive mitochondrial fusion. As a result, the activity of mitochondrial respiratory chains is reduced, which leads to severe cell defects (213–215). On the other hand, poisoning promotes apoptosis by promoting DRP1 phosphorylation, subsequentially leading to the fission of mitochondria in cardiac cells (216).

#### Mitophagy and Poisoning

Doxorubicin (DOX), organophosphorus, nicotine, excessive alcohol, and other toxic substances can also inhibit the expression of Parkin, deteriorate the mitophagy ability of cardiac cells, damage mitochondria, and destroyed substances in cardiac cells will continue to accumulate, causing cardiac damage (26, 217, 218). Moreover, the decrease of mitophagy can lead to excessive ROS in cardiac cells, further promote the release of cytochrome c and cysteine aspartate protease, disrupt the stability of mitochondria DNA, inhibit the activity of respiratory electron-transport chain, and reduce both oxygen utilization and consumption. It can even initiate mitochondrial apoptosis and induce mitochondrial damage (26, 88, 219–224). Parkin overexpression increases mitophagy, which aggravates cell death through poisoning. And, Parkin knockdown has the opposite effect (225, 226). DOX can also dysregulate the cytosolic and mitochondrial signaling axes, which leads to mitophagy destruction and arrhythmias, causing impaired mitochondrial clearance, the accumulation of dysfunctional mitochondria, ROS overload, and a lack of Adenosine triphosphate (ATP). Meanwhile, DOX can also phosphorylate BNIP3 and then inhibit mitophagy, which is closely related to the mitochondrial sirtuins (SIRT3-SIRT4) pathway (26, 27, 150, 206, 207, 221, 225, 227, 228). Recently, it was reported that excessive DOX can also significantly induce elevated insulin-like growth factor-II receptor (IGF-IIR) expression, IGF-IIR induces myocardial hypertrophy and cardiomyocyte death in a paracrine/autocrine manner. Concurrently, IGF-IIR can further promote mitophagy by inducing Parkin expression and cause cardiac damage (28, 229, 230).

## Therapeutic Application of Mitochondrial Dynamics and Mitophagy

Mitochondrial dynamics and mitophagy play an important role in cardiotoxicity, so they can be regarded as potential therapeutic targets. Cardiotoxicity can be treated, or its progress can be delayed by promoting or inhibiting mitochondrial dynamics and mitophagy, thus maintaining the functional stability of mitochondria and reducing cell damage under the influence of injury factors (Table 1).

**Table 1:** Therapeutic application of mitochondrial dynamics and mitophagy.

**Injury factors**	**Key protein**	**Representative interventions**	**Mechanisms**	**Effects to cardiotoxicity**
Hypoxia	FUNDC1	Hypoxia preconditioning	Mitophagy	Protection
Hypoxia	DRP1	Schaftoside	Fission	Protection
OS	PINK1	Zine, Melatonin	Mitophagy	Protection
OS	BNIP3	Acute exercise	Mitophagy	Protection
OS	DRP1	Melatonin	Fission	Protection
Hyperglycemia	DRP1	Metformin	Fission	Protection
Hyperglycemia	MFN1	Metformin	Fission	Protection
Anthracyclines poisoning	BNIP3	Acute exercise	Mitophagy	Protection
Organophosphate poisoning	Parkin	Salidroside	Mitophagy	Protection
Nicotine poisoning	OPA1	MYLS22	Fusion	Protection

Hypoxia preconditioning induced FUNDC1-dependent activation of mitophagy and decreased I/R-induced cardiac injury (79). Shaftaside is a natural flavonoid. Shaftaside and MYLS22 can effectively inhibit the expression of DRP1 and OPA1 to inhibit mitochondrial fission and reduce the cardiotoxicity induced by hypoxia, oxidative stress, hyperglycemia and poisoning (231). In healthy and MIRI rat cardiomyocytes, Zn and salidroside can activate mitophagy by up-regulating the expression of PINK1/Parkin, clear damaged mitochondria, and maintain normal cardiac function (86, 232–234). Myocardial mitochondrial function adapts to stress during acute exercise and manifests as significant upregulation of the mitophagy-related protein BNIP3, which stimulates mitophagy and minimizes myocardial injury. Melatonin and acute exercise preconditioning can activate the expression of PINK1 and BNIP3, respectively, to enhance mitophagy and decreased ROS-induced cardiac injury (235–242). Metformin can inhibit mitochondrial fission by the activation of MFN1 and the inhibition of DRP1, which decreased hyperglycemia -induced cardiotoxicity (176, 243–247).

## Conclusions and Prospects

Mitochondrial dynamics and mitophagy are decisive factors for maintaining the homeostasis of the cardiac cell environment and ensuring the normal function of the cardiac. Hypoxia, hyperglycemia, and oxidative stress mainly interfere with mitochondrial fission and mitophagy to cause cardiotoxicity, while poisoning mainly interferes with mitochondrial fusion and mitophagy to cause cardiotoxicity. In view of different injury factors, taking different representative interventions to maintain the normal mitochondrial dynamics and mitophagy is of great significance for the prevention and treatment of cardiotoxicity. However, whether different therapeutic effects can be achieved through different routes of administration requires further research. Therefore, the exploration, regulation, and monitoring of the balance point in mitochondrial dynamics is crucial for preventing external injury factors from inducing cardiotoxicity. However, at the advent, the specific mechanism of action of mitochondrial dynamics and mitophagy in the process of cardiotoxicity is yet to be established. Extensive empirical studies are needed to study, confirm, and provide a theoretical basis for mitochondrial dynamics-induced cardiotoxicity, which would help prevent various causes of cardiotoxicity.

## Author Contributions

HS and YY contributed to conception and design of the study. YY wrote the first draft of the manuscript. All authors contributed to the article and approved the submitted version.

## Funding

This study was funded by Science Foundation of Liaoning Education Department (FWZR2020012), 345 Talent Project of Shengjing Hospital, Science and Technology Department of Liaoning Province (2020JH2/10300126).

## Conflict of Interest

The authors declare that the research was conducted in the absence of any commercial or financial relationships that could be construed as a potential conflict of interest.

## Publisher's Note

All claims expressed in this article are solely those of the authors and do not necessarily represent those of their affiliated organizations, or those of the publisher, the editors and the reviewers. Any product that may be evaluated in this article, or claim that may be made by its manufacturer, is not guaranteed or endorsed by the publisher.
